# Egg Yolk-Free Vegan Mayonnaise Preparation from Pickering Emulsion Stabilized by Gum Nanoparticles with or without Loading Olive Pomace Extracts

**DOI:** 10.1021/acsomega.2c02149

**Published:** 2022-07-20

**Authors:** Alican Akcicek, Salih Karasu, Fatih Bozkurt, Selma Kayacan

**Affiliations:** † Faculty of Chemical and Metallurgical Engineering Department of Food Engineering, Yildiz Technical University, Davutpasa Campus, Esenler, Istanbul 34210, Turkey; ‡ Faculty of Tourism Department of Gastronomy and Culinary Arts, Kocaeli University, Kartepe, Kocaeli 41080, Turkey; § Engineering and Architecture Faculty, Department of Food Engineering, Muş Alparslan University, Güzeltepe, Muş 49250, Turkey

## Abstract

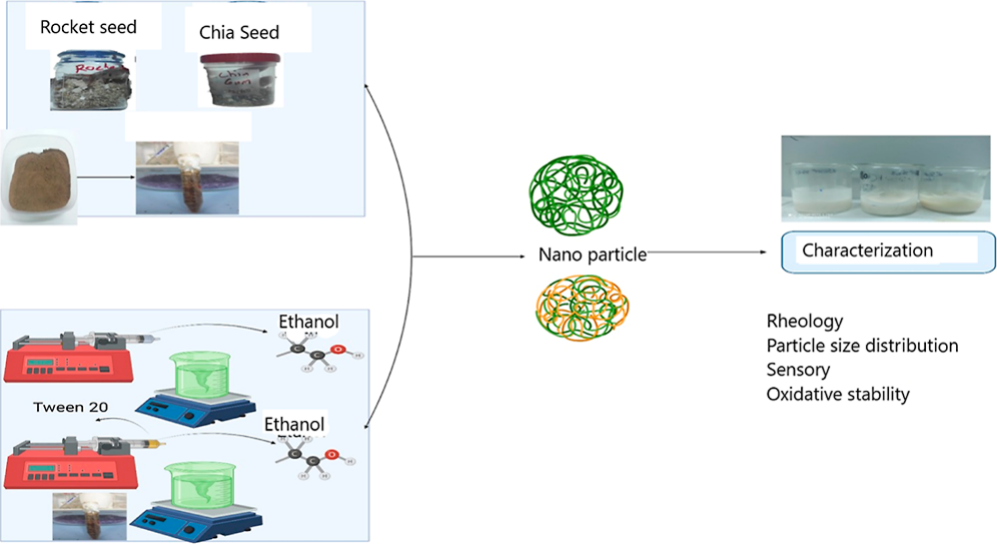

The yolk-free mayonnaise was formed by Pickering emulsions stabilized by free and encapsulated olive pomace extracts (OPEs) in rocket seed [rocket seed gum nanoparticle (RSGNP)] and chia seed gum nanoparticles at different nanoparticle concentrations. The yolk-free mayonnaise and the control mayonnaise samples were compared in terms of appearance, microstructural, droplet size, emulsion stability, rheological, oxidative stability, and sensory properties. The droplet size decreased by increasing the nanoparticle concentration in yolk-free mayonnaise samples. The yolk-free mayonnaise samples prepared with OPE-loaded gum nanoparticle showed shear-thinning, solid-like and recoverable characteristics, which increased as the increase in the nanoparticle concentration. The emulsion stability and capacity increased by increasing the nanoparticle concentration in the yolk-free mayonnaise samples. OPE-loaded gum nanoparticle-stabilized yolk-free mayonnaise samples exhibited higher IP (induction period) values than the control samples. OPE–RSGNP 1% mayonnaise was observed to be the closest sample to the control sample with its sensory properties, general acceptability, and similar microstructural and rheological properties. The results of this study indicated that Pickering emulsions stabilized by gum nanoparticles could be used as healthy alternatives to the egg yolk in conventional mayonnaise.

## 1 Introduction

Mayonnaise is an oil-in-water emulsion and is composed of 65–80% oil, 6–20% egg yolk, and 3–5% vinegar.^1^ Egg yolk plays a critical role in the stability and structural properties of mayonnaise due to having emulsifying properties, reducing surface tension, and increasing emulsion stability.^2^ Nevertheless, egg yolks have high cholesterol levels and saturated fatty acids, which caused obesity^3^ and are open to microbial contamination by *Salmonella enteritidis*.^4,5^ In this sense, studies are focused on founding the potential novel emulsifier for mayonnaise. Adding novel emulsifiers to egg yolk-free mayonnaise formulation is getting attracted by the customer due to consumer preference increase toward veganism.^2^ Once the preparation of the mayonnaise formulation, the quality of the mayonnaise depends on the properties such as pH, emulsion stability, appearance, droplet size, and rheological properties.^6^ It is the challenge of the task to answer the consumer of the needs due to an important difference of the quality between egg yolk-based mayonnaise and mayonnaise-like emulsions. Therefore, quality properties of the egg yolk-free mayonnaise such as microstructure, rheology, textural, stability, and sensory should be considered providing characteristics of egg-based mayonnaise. The forming of the balance of these properties ensures the consumer of needs and admission of the mayonnaise instead of the egg-based mayonnaise.^3^

Nowadays, Pickering emulsions are used for the enhancement of egg yolk-free mayonnaise, and some studies were reported in the literature.^3,4,7^ Pickering emulsion utilized solid particles to provide stabilization contrary to the conventional surfactants and emulsifiers. The mechanisms of stabilization are different for conventional and Pickering emulsions such as electrostatic stabilization, surface tension, and steric stabilization of reduction with the help of surfactants or polymeric molecules in the conventional emulsion.^8^ Studies recommended that gums and plant-based proteins could be utilized as an emulsifier and stabilizer source in conventional emulsion instead of egg yolk.^2,9,10^ While adsorption of traditional emulsifiers and macromolecules are mostly reversible in the conventional emulsions, adsorption of the solid particles in the interface is considered irreversible for Pickering emulsions.^8^ The solid particles are settled to the oil–water interface creating obstacle barrier, to avoid aggregation between droplets, and prevent the probable flocculation, coalescence, and sedimentation.^11^ The Pickering emulsions have higher stability, smaller size distribution than the conventional emulsions.^7^ In addition, Pickering emulsions demonstrated better and higher stability against coalescence and Ostwald deripening.^12^

The food-grade Pickering emulsifiers are classified into four groups, inorganic particles, carbohydrate particles, lipid particles, and protein particles.^4^ Inorganic and synthetic particles have been utilized for stabilizing Pickering emulsion, while organic and natural food grade Pickering emulsifiers and stabilizers such as natural plant-based carbohydrate and protein particles gained much more attention for food applications.^13^ Carbohydrate particles have limitations such as poor surface activity and emulsification work. However, to overcome these challenges, some techniques improved such as surface coating with protein particles, surface modification, and physical modification like ultrasound and grinding.^8,14^ Carbohydrate particles such as chitosan, cellulose, and starch nanoparticles are the most known and used particles as Pickering stabilizers.^15−17^ Nanoparticles could be utilized as an emulsifier for stabilizing the Pickering emulsions instead of the conventional emulsifiers. Nanoparticles are distributed in the oil or water phase and dissolve in one phase and act as an emulsifier.^11^ For instance, gum nanoparticles could be used as a Pickering stabilizer due to the model emulsion system formed, and oxidative stability of emulsion increased when the olive pomace extract (OPE)-loaded gum nanoparticles were incorporated into the Pickering emulsion.^18^ Olive pomace is a byproduct that contains various bioactive compounds, especially phenolic compounds that emerge during olive oil production.^19^ The OPE contains hydroxytyrosol tyrosol and luteolin, which have good antioxidant, anti-inflammatory, and antimicrobial properties.^20^ The Pickering emulsion stabilizer of the blank and OPE-loaded gum nanoparticles in the mayonnaise not only provides a good source of olive pomace phenolic compounds but is also useful for human health and utilized for food applications.^20^ These added-value compounds are utilized to develop nutraceuticals and functional food ingredients.^21^ However, there are no reports about rocket seed gum (RSG) and chia seed gum nanoparticles (CSGNPs), OPE-loaded RSG, and CSGNPs, which can be used as a Pickering emulsion stabilizer in the egg yolk-free mayonnaise. Therefore, the aim of this study is evaluating the blank and OPE-loaded gum nanoparticles as a Pickering emulsion stabilizer instead of egg yolk in the mayonnaise. The physicochemical and rheological properties of the egg-yolk-free mayonnaise were evaluated contrary to control mayonnaise. The results provide information about the importance of pomace and plant-based natural gums for the food industry.

## 2 Materials and Methods

### 2.1 Materials

Olive pomace was supplied from the Ekin Kocadağ Food Industry. Olive pomace was dried at 50 °C for 7 h. Then, kernels were removed from dried bulk and grounded to olive pomace powder by using mill flour. Rocket seed and chia seed were acquired from local producers. The sunflower oil, vinegar, salt, and sugar were acquired by local markets. All reagents used were of analytical grade.

### 2.2 Methods

#### 2.2.1 Preparations of the Olive Pomace Extract

Before extraction, olive pomace powder was washed three times by utilizing hexane to remove olive oil. The OPE was prepared by using 80% methanol/water. The method used in this study was given in our previous study.^22^

#### 2.2.2 Preparations of Gum Solution

The gum solutions with a concentration of 0.1% were prepared by our previous study.^18^ First, the gum dissolved in distilled water for 2 h at 500 rpm at room temperature and then left overnight to complete hydration at 4 °C. After the dissolution of gum, centrifugation was applied to the solution to remove impurities. The pH values of the gum solutions were adjusted to 8 and 7 for RSG and CSG, respectively (HI 2211, UK) by using 0.1 N NaOH.

#### 2.2.3 Production of Nanoparticles

The desolvation technique was used to fabricate gum nanoparticles. The gum nanoparticles were prepared through using a desolvation method by dropwise addition of the desolvating agent (ethanol) continuously. The modified version of the method described by Taheri et al.^23^ was used in this study.^22^ The gum solutions (solvent) were blended at 800 rpm for 5 min. OPE (0.1%) and Tween 20 (0.5%) were added in optimized amounts of ethanol (antisolvent). Tween 20 was used for the better dissolving of OPE in ethanol. Ethanolic OPE (0.5 mL/min) was put dropwise to the gum solution (solvent phase) using a syringe pump system (New Era, NE, USA). After adding the ethanol, the solution was stirred at 800 rpm for 10 min. Then, ultrasonication (Hielscher UIP1000hdT, Germany) of 100 W was performed to the solutions for 1 min (every 30 s wait for 10 s) in an ice bath. The nanoparticle suspensions were centrifuged at 9000 rpm for 30 min. Nanoparticles were redispersed with 5 mL of distilled water and then freeze-dried without using cryoprotectants. The same experimental analysis without OPE and Tween 20 was performed for blank gum nanoparticle fabrication.

#### 2.2.4 Preparation of the Egg Yolk-Free Mayonnaise

The egg yolk-free mayonnaise was prepared by using the method.^4^ Aqueous nanoparticle solutions were composed of xanthan gum (0.4%, m/v), sugar (4%, m/v), salt (2%, m/v), vinegar (10%, m/v), OPE, RSG nanoparticle (RSGNP), CSGNP, OPE–RSGNP, OPE–CSGNP (1, 2, 3%, m/v), and some deionized water. The oil-in-water emulsion was acquired by blending aqueous nanoparticle solutions (10 mL) and sunflower oil (10 mL). These Pickering emulsions were emulsified by using a high-speed shear homogenizer at 10,000 rpm for 2 min. The appearance image of Pickering emulsions was taken at 24 h and 30 days. The samples were kept closed and stored at temperature of 25 °C. Sodium benzoate was put in the Pickering emulsions to avoid microbial growth. At last, the nanoparticle concentrations in the mayonnaise determined 0.5, 1, 1.5%. The formulation of the mayonnaise samples is given in Table 1.

**Table 1:** Formulation of the Mayonnaise Samples^a^

ingredients	control	RSGNP	CSGNP	OPE–RSGNP	OPE–CSGNP
aqueous phase (% v)	50	50	50	50	50
sunflower oil (% v)	50	50	50	50	50
aqueous phase (% m/v)	100	100	100	100	100
vinegar (m)	10	10	10	10	10
salt (m)	2	2	2	2	2
sugar (m)	4	4	4	4	4
sodium benzoate (m)	0.3	0.3	0.3	0.3	0.3
xanthan gum (m)	0.4	0.4	0.4	0.4	0.4
NP concentrations (m)		1–2–3	1–2–3	1–2–3	1–2–3
egg yolk powder (m)	14				

a RSGNP: rocket seed gum nanoparticle, CSGNP: chia seed gum nanoparticle, OPE–RSGNP: olive pomace extract-loaded RSGNP, and OPE–CSGNP: olive pomace extract-loaded CSGNP.

#### 2.2.5 Droplet Size Analysis and Microstructure

The droplet size of emulsions after 24 h and 30 days of storage was determined. First, a drop of different emulsions was gently poured onto a glass slide and then photographed using a light microscope (Olympus, JAPAN) equipped with a digital camera. To estimate the average size of emulsion droplets, three images were taken from each sample, and then, Olympus software was performed by counting at least 30 droplets in different images. The optical image of the Pickering emulsions was detected using a microscope (Olympus, JAPAN). The Pickering emulsions were added to the middle of the glass slide and images obtained at 10× magnification.

#### 2.2.6 Rheological Properties

##### 2.2.6.1 Steady Shear Properties

All rheological analyses were performed by a stress and temperature-controlled rheometer (Anton Paar MCR 302, Graz, Austria). Rheological analysis was performed at 25 °C and 0.5 mm gap interval.

Steady shear analyses were carried out in the range of shear rate 0.1–100 s^–1^. The acquired data were fitted to the power law model eq 1
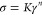
1where σ specifies shear stress (Pa), *K* indicates consistency index, γ indicates shear rate (s^–1^), and *n* indicates flow behavior index.

##### 2.2.6.2 Dynamic Frequency Properties

Dynamic frequency analyses were performed in low shear stress at constant strain to determine storage and loss modulus. The analysis was performed at 0.1% strain with an angular frequency of 0.1–62.8 rad/s. Dynamic frequency rheological parameters were specified using the power law model and nonlinear regression^24^
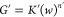
2
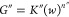
3where *G*′ and *G*″ are storage and loss modulus, *K*′ and *K*″ specify consistency index values, and *n*′ and *n*″ are dependence degree of storage modulus and the loss modulus to frequency.

##### 2.2.6.3 Three Interval Thixotropic Test (3-ITT)

The three interval thixotropic test (3-ITT) was provided to get information about the recovery of the samples after deformation was applied. In the first time interval, the emulsion samples in the linear viscoelastic region (LVR) were exposed to a low shear rate value (0.5 s^–1^) for 50 s. In the second time interval, emulsion samples in the non-LVR were exposed to a high shear rate value (150 s^–1^) for 30 s. The third time interval in the LVR was exposed to a low shear rate value (0.5 s^–1^) for 50 s. The alter of the viscoelastic matrix of the emulsion samples was observed.

The previous method^25^ used to evaluate the 3-ITT parameters about deformation (% *D*_R_) was performed using eq 4, and recovery percentage of the emulsion samples at 30 s after the deformation applied was performed using eq 5 (% Rec_30_).
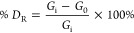
4
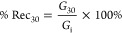
5where *G*_i_ and *G*_0_ stated that *G*′ values of emulsion samples at the first state0 and after the deformation applied, respectively. *G*_30_ indicated *G*′ values of emulsion samples at initial 30 s after deformation.

### 2.7 Emulsion Appearance, Capacity, and Stability

The bulk appearance of the emulsion was taken with the smartphone camera (Xiaomi Note 8 Pro, CHINA). Emulsion capacity was determined by the emulsion layer height of 24 h divided by the total height of fresh emulsions, and emulsion stability was determined by the percentage of the emulsion layer after 30 days to 24 h.^4^

### 2.8 Oxidative Stability

Yolk-free mayonnaise samples which stabilized OPE-loaded gum nanoparticles were analyzed by utilizing the oxidative tester (Velp Scientifica, Usmate, MB, Italy). A 20 g of Pickering emulsion was weighed into the sample cells homogeneously. The temperature of the oxidative tester was adjusted to 90 °C and the oxygen pressure to 6 bar. Oxidative stability values of the mayonnaise samples were determined as the induction period. The induction period was used to evaluate the oxidative stability of the mayonnaise samples.

### 2.9 Sensory Analysis

The sensory properties of mayonnaise samples were evaluated based on a five-point hedonic test by 30 semi-trained panelists, which included academicians and students.^26^ The following sensory attributes were assessed: appearance, color, taste, spreadability, texture, and overall acceptability. Before analysis, the panelists were briefly informed about scales and sensory attributes. All mayonnaise samples were numbered by three-digit numbers randomly. The mayonnaise samples were randomly served to the panelists in white plastic dishes with teaspoons. The water was used for cleaning the mouth between different mayonnaise samples. The ranking was defined as follows 1 = the lowest and 5 = the highest.

### 2.10 Statistical Analysis

All analyses were carried out in triplicate, and data were given mean ± standard deviation. Statistical analyzes were evaluated by one-way ANOVA (Tukey test) using Minitab14. Statistical significance was determined as *p* < 0.05. The results of the rheological analysis were fitted to the power law model with the assistance of the non-linear regression and evaluated the model applicability by the coefficient of determination (*R*^2^). The model parameters of the steady shear rheological properties of Pickering emulsion and dynamic frequency nonlinear regression analyses were evaluated by using the Statistica software program (StatSoft, Inc., Tulsa, OK).

## 3 Results and Discussion

### 3.1 Droplet Size and Microstructure Properties of the Yolk-Free Mayonnaise

**Figure 1:**
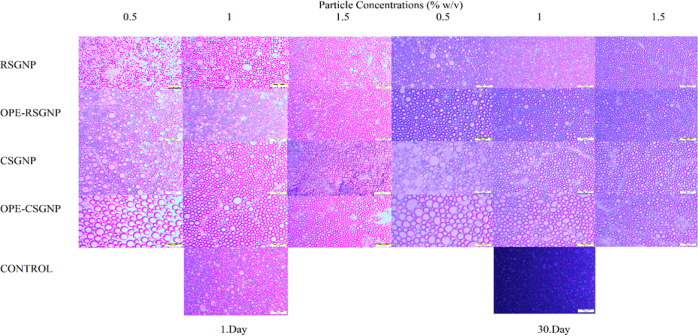
Microstructural properties of the mayonnaise samples RSGNP: rocket seed gum nanoparticle, CSGNP: chia seed gum nanoparticle, OPE–RSGNP: olive pomace extract-loaded RSGNP, and OPE–CSGNP: olive pomace extract-loaded CSGNP.

The droplet sizes of mayonnaise samples are presented in Table 2. The droplet size decreased as increasing nanoparticle concentration in yolk-free mayonnaise samples. With this result, it could be explained that higher concentration of gum nanoparticles in aqueous solution showed larger surface and interfacial area, hindering the coalescence.^27,28^ A similar result was given in references (29). (30), Blank and OPE-loaded RSGNPs of yolk-free mayonnaise samples at 1.5% nanoparticle concentration and control samples showed no significant difference (*p* < 0.05). In addition to the stability, mayonnaise or salad dressing with a lower droplet size displayed well delicious taste and better mouthfeel.^4,31^ Also, OPE–RSGNP had a lower droplet size than the OPE–CSGNP mayonnaise samples. This result could be explained by RSG having a lower molecular weight and high protein content.^18^ The microstructural properties of all mayonnaise samples are presented in Figure 1. All mayonnaise samples showed spherical droplets. As can be seen in Figure 1, an increase in the nanoparticle concentration led to a decrease in the smaller droplet size, which is consistent with droplet size results. In addition, mayonnaise samples with 1 and 1.5% nanoparticle concentration and control mayonnaise showed similar structures and the highest homogeneity in the droplet size. As the nanoparticle concentration in the emulsion increases, the droplet size of the emulsion decreases. Therefore, these results suggested that the microstructural properties of the emulsion could be improved by the addition of nanoparticles.

**Table 2:** Emulsion Capacity, Emulsion Stability and Droplet Diameter of the Mayonnaise Samples^a^

Run	NP concentration (%)	emulsion capacity (%)	emulsion stability (%)	*D*_1_ (μm)	*D*_30_ (μm)
RSGNP	0.5	89.64 ± 0.50f	92.03 ± 0.04f	31.3 ± 1.15c	33.53 ± 1.87de
RSGNP	1	91.11 ± 0.52e	94.67 ± 1.13cde	32.43 ± 1.07c	28.66 ± 1.25f
RSGNP	1.5	98.16 ± 0.23b	99.04 ± 0.57ab	22.16 ± 2.33de	25.23 ± 0.98g
OPE–RSGNP	0.5	96.55 ± 0.12d	97.31 ± 0.34c	36.53 ± 2.95bc	42.63 ± 1.21c
OPE–RSGNP	1	98.37 ± 0.20b	97.66 ± 0.09c	26.53 ± 2.89d	29.03 ± 0.87f
OPE–RSGNP	1.5	98.55 ± 0.18b	98.31 ± 0.41b	22.7 ± 0.92e	26.23 ± 2.38fg
CSGNP	0.5	91.53 ± 0.28e	95.26 ± 0.52d	53.9 ± 3.15a	58.6 ± 4.59b
CSGNP	1	91.62 ± 0.53e	95.87 ± 0.02d	37.23 ± 2.50b	36.43 ± 3.21d
CSGNP	1.5	97.57 ± 0.29c	99.34 ± 0.14a	33.5 ± 1.19c	38.93 ± 4.18cd
OPE–CSGNP	0.5	96.74 ± 0.22d	92.01 ± 0.09f	55.3 ± 1.35a	79.06 ± 1.84a
OPE–CSGNP	1	97.36 ± 0.19c	93.27 ± 0.62e	37.96 ± 1.90b	54.1 ± 2.40b
OPE–CSGNP	1.5	98.77 ± 0.19b	99.64 ± 0.26a	26.96 ± 1.45d	35.9 ± 2.70d
Control	0	99.55 ± 0.07a	99.71 ± 0.10a	20.63 ± 2.80e	25.2 ± 4.35fg

a RSGNP: rocket seed gum nanoparticle, CSGNP: chia seed gum nanoparticle, OPE–RSGNP: olive pomace extract-loaded RSGNP, and OPE–CSGNP: olive pomace extract-loaded CSGNP. Lowercase letters indicate the relationship between all mayonnaises. Values that do not share the same letter differ significantly (*p* < 0.05).

### 3.2 Rheological Properties

#### 3.2.1 Steady Shear Properties

**Figure 2:**
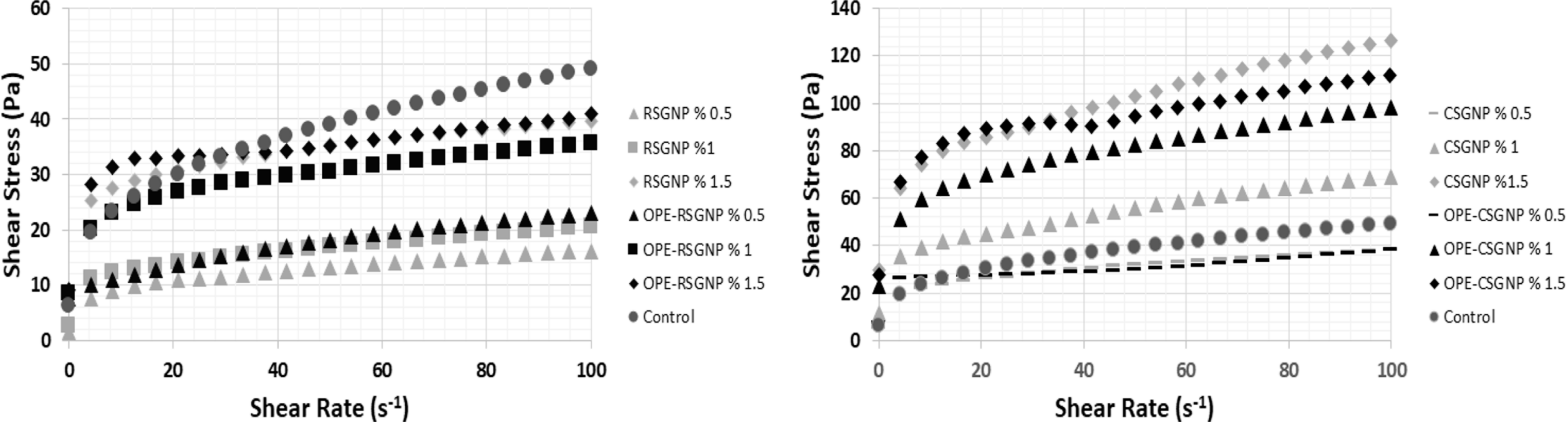
Steady shear rheological properties of mayonnaise.

The steady shear properties of all mayonnaise are presented in Figure 2. As can be seen in Figure 2, the viscosity of all mayonnaises decreased with an increase in the shear rate. Meaning that control and yolk-free mayonnaise samples showed pseudoplastic flow character. Emulsion droplets were located on the flow layer in the shear direction, and oil droplet agglomeration was separated into small droplets.^3^ The applied shear stress deformed to the Pickering emulsion system and resulted in the accelerated shear rate, and quicker deformation of the emulsion led to the reduction of the viscosity.^4^

The consistency index (*K*) and flow behavior index (*n*) values were calculated by using the power law model and are given in Table 3. All mayonnaise samples displayed non-Newtonian and shear thinning behavior (*n* < 1). All mayonnaise showed *n* < 1, indicating the pseudoplastic nature of mayonnaise.^2,3,29,32,33^ The *K* value is the factor that specified the viscous nature of the fluid, and the higher *K* value stated the strong emulsion structure.^34,35^ Generally, the lower *K* value stated that the emulsion had low viscosity.^4,31^ The less value of *n* indicated the strongest shear thinning behavior.^36^

**Table 3:** Power Law Model Parameters of Mayonnaise Samples^a^

Run	NP concentration (%)	*K* (Pa s^n^)	*n*	*R*^2^
RSGNP	0.5	4.85 ± 0.62i	0.25 ± 0.008	0.99
RSGNP	1	7.00 ± 1.02h	0.22 ± 0.0002	0.99
RSGNP	1.5	18.21 ± 0.60e	0.17 ± 0.004	0.99
OPE–RSGNP	0.5	9.09 ± 1.10h	0.34 ± 0.006	0.99
OPE–RSGNP	1	13.91 ± 1.68g	0.20 ± 0.02	0.99
OPE–RSGNP	1.5	20.8 ± 0.19d	0.14 ± 0.004	0.97
CSGNP	0.5	12.92 ± 0.12g	0.23 ± 0.002	0.99
CSGNP	1	21.70 ± 0.67d	0.24 ± 0.002	0.99
CSGNP	1.5	37.21 ± 1.34b	0.22 ± 0.01	0.98
OPE–CSGNP	0.5	16.80 ± 0.30f	0.16 ± 0,008	0.94
OPE–CSGNP	1	37.41 ± 1.41b	0.20 ± 0,0002	0.99
OPE–CSGNP	1.5	51.99 ± 0.09a	0.16 ± 0.009	0.98
Control	0	12.01 ± 1.14g	0.30 ± 0.01	0.99

a RSGNP: rocket seed gum nanoparticle, CSGNP: chia seed gum nanoparticle, OPE–RSGNP: olive pomace extract-loaded RSGNP, and OPE–CSGNP: olive pomace extract-loaded CSGNP. Lowercase letters indicate the relationship between all mayonnaises. Values that do not share the same letter differ significantly (*p* < 0.05).

The *K* values of the OPE–RSGNP and OPE–CSGNP were higher than RSGNP and CSGNP mayonnaise samples, respectively, and differences were found to be statistically significant (*p* < 0.05). A similar result was reported.^37^ In nature, plant polyphenols are often closely associated with polysaccharides as they both contain large amounts of hydrophilic groups and hydrophobic groups, so they can be complexed or cross-linked with polysaccharides.^38−40^ Due to the polysaccharide composition of dried olive pomace, pomace can be a potential source for gelling pectic material.^41^ Rocket seed and CSGNPs can bind to olive pomace polyphenols due to hydrogen bonding and hydrophobic interactions.

Gums and olive pomace polyphenols form hydrogen bonds very easily due to the containing high amount of hydroxy groups. In addition, rocket seed and chia seed gum contain sugar rings and interact with the hydrophobic groups of olive pomace such as luteolin. A strong network is formed between them as a result of hydrogen bonds and hydrophobic interactions. Therefore, as the concentration of OPE–RSGNP and CSGNP increases, the *K* values of the yolk-free-based mayonnaise samples increase. The high amount of the phenolic content caused a further increase in viscosity. A similar result was obtained.^40,42^ OPE–CSGNP 1.5% displayed stronger shear thinning behavior (*n* < 1) with a higher *K* value among all mayonnaise samples (*p* < 0.05). In addition, an increase in the nanoparticles concentration in the mayonnaise led to a higher *K* value. The high amount of gum nanoparticles stabilized a larger surface area to provide the highest viscosity with high-molecular weight and long-chain branch structure.^35,43^ Also, the more gum nanoparticles provide excellent resistance to droplet movement, preventing the coalescence led to the smaller oil droplets.^35^ The results were consistent with droplet size analysis. Moreover, the increasing OPE content for OPERSGNP and CSGNP-stabilized yolk-free mayonnaise with higher OPE led to the higher consistency index (*K*), suggesting the creation of a stronger network structure between droplets (Zhang et al., 2020) which contributed to the high viscosity of the emulsion.^40^ The differences in the *K* value of the control mayonnaise and yolk-free mayonnaise samples were related to the interaction of the emulsion droplets, the strength of the network matrix, and the droplet size of the emulsions.^4^ The *K* value of the control mayonnaise and OPE–RSGNP 1% and CSGNP 0.5% mayonnaise samples showed no significant differences (*p* < 0.05). The other yolk-free mayonnaise samples and control mayonnaise samples of *K* value showed significant differences (*p* < 0.05). However, the higher *K* value does not mean a higher viscosity; also the *n* value and other parameters are considered in this sense.^4,36^ The *K* value and higher *n* value of the control mayonnaise samples were mainly related to the egg yolk, which acted as the thickening agent as an emulsifier.^44^

#### 3.2.2 Viscoelastic Properties

**Figure 3:**
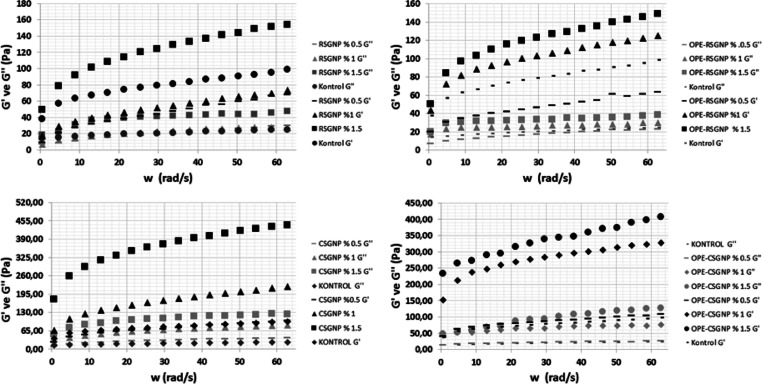
Dynamic rheological properties of mayonnaise.

The viscoelastic properties of all mayonnaise samples are illustrated in Figure 3. Mayonnaise could be considered a gel-like structure.^6^ For control and yolk-free mayonnaise samples, *G*′ values higher than the *G*″ values whole frequency range indicate that the behavior of the Pickering emulsion was a dominantly solid elastic character.^3,30,32^ In addition, the result recommended that control mayonnaise samples and egg yolk-free mayonnaise samples produced at all nanoparticle concentrations exhibited the viscoelastic structure expected from the desired mayonnaise. The result was associated with a three-dimensional network structure that occurred by the interaction between droplets.^4,12^ In the LVR, *G*′ and *G*″ values increased with increasing nanoparticle concentrations, indicating that elastic dominant behavior with gel-like properties gradually increased at higher concentrations of gum nanoparticles. Also, gum nanoparticles that contained OPE have higher *G*′ values than the RSGNP and CSGNP mayonnaise samples. Gum nanoparticles bridged between oil droplets could improve the strongest interaction between the oil droplets.^3^ A similar gel-like structure was reported in references (3). (4)(28), Both *G*′ and *G*′ increased progressively with the increase in the OPE content in the OPERSGNP and CSGNP, suggesting that the network structure becomes more cohesive, compact, and stronger for OPERSGNP and CSGNP-stabilized yolk-free mayonnaise.^40^ The OPE RSGNP and OPECSGNP with 1 and 1.5%, RSGNP with 1.5%, and CSGNP with 1 and 1.5% samples have higher *G*′ and *G*″ values than the control samples, which indicated that higher particles led to an increase in the solid character. The results obtained from the frequency sweep test suggested that the spreadability properties of mayonnaise samples can be estimated. The improvement in solid-like structure with the increase in the nanoparticle concentration shows that the spreadable properties of mayonnaise samples can be improved without egg yolk.

The viscoelastic parameters of the mayonnaise samples were calculated by using the power-law model (Table 4). The *K*′ value was found higher than the *K*″ values for all mayonnaise samples, which indicated that the elastic solid character was dominant on the viscous character (*R*^2^ = 0.99). An increase in the nanoparticle concentration led to an increase in the higher *K*′ and *K*″ values due to more particles located on oil droplets and led to formed three-dimensional network structures with high gel strength. The OPE RSGNP, OPECSGNP with 1 and 1.5%, RSGNP with 1.5%, and CSGNP with 1 and 1.5% samples have higher *K*′ and *K*″ values than the control samples, and significant differences were observed (*p* < 0.05). The results were consistent with the *G*′ and *G*″ values of mayonnaise samples. *n*′ and *n*″ values showed frequency dependence of the *G*′ and *G*″ values. Their values affected emulsion transportation and canning in the product application in the industry.^3,4^ The control sample of the *n*′ and *n*″ values showed no significant differences with that of OPE–RSGNP with 1% and OPE–CSGNP with 1.5%, respectively. This could be the interaction of droplets and the internal matrix of the mayonnaise.^3,4^

**Table 4:** Power Law Model Parameters for Dynamic Rheological Properties of Mayonnaise Samples^a^

run	NP concentration (%)	*K*′ (Pa s^n^)	*n*′	*R*^2^	*K*″ (Pa s^n^)	*n*″	*R*^2^
RSGNP	0.5	9.20 ± 0.22k	0.475 ± 0.02	0.99	4.83 ± 0.72i	0.43 ± 0.05	0.95
RSGNP	1	15.66 ± 1.06j	0.36 ± 0.01	0.99	7.60 ± 1.08h	0.31 ± 0.02	0.99
RSGNP	1.5	52.16 ± 1.18f	0.24 ± 0.007	0.99	19.40 ± 0.41f	0.21 ± 0.003	0.98
OPE–RSGNP	0.5	18.25 ± 2.63j	0.29 ± 0.01	0.97	5.98 ± 1.31hi	0.32 ± 0.001	0.98
OPE–RSGNP	1	49.66 ± 0.19g	0.21 ± 0.001	0.99	18.74 ± 0.29f	0.10 ± 0.003	0.96
OPE–RSGNP	1.5	57.93 ± 0.94e	0.22 ± 0.002	0.99	22.53 ± 0.33e	0.11 ± 0.007	0.96
CSGNP	0.5	27.68 ± 3.43i	0.32 ± 0.02	0.99	12.03 ± 1.56g	0.29 ± 0.02	0.99
CSGNP	1	67.06 ± 1.70d	0.28 ± 0.001	0.99	27.15 ± 0.85d	0.27 ± 0.01	0.99
CSGNP	1.5	181.54 ± 0.08b	0.2 ± 0.001	0.99	46.37 ± 0.31b	0.18 ± 0.004	0.99
OPE–CSGNP	0.5	41.78 ± 0.04h	0.21 ± 0.002	0.99	13.47 ± 0.34g	0.16 ± 0.01	0.96
OPE–CSGNP	1	162.73 ± 1.71c	0.16 ± 0.007	0.99	41.07 ± 1.99c	0.14 ± 0.01	0.94
OPE–CSGNP	1.5	209.13 ± 8.96a	0.14 ± 0.004	0.99	61.76 ± 0.97a	0.16 ± 0.002	0.96
control	0	39.51 ± 2.75h	0.21 ± 0.003	0.99	12.32 ± 0.57g	0.16 ± 0.004	0.95

a RSGNP: rocket seed gum nanoparticle, CSGNP: chia seed gum nanoparticle, OPE–RSGNP: olive pomace extract-loaded RSGNP, and OPE–CSGNP: olive pomace extract-loaded CSGNP. Lowercase letters indicate the relationship between all mayonnaises. Values that do not share the same letter differ significantly (*p* < 0.05).

#### 3.2.3 3-ITT Rheological Properties

**Figure 4:**
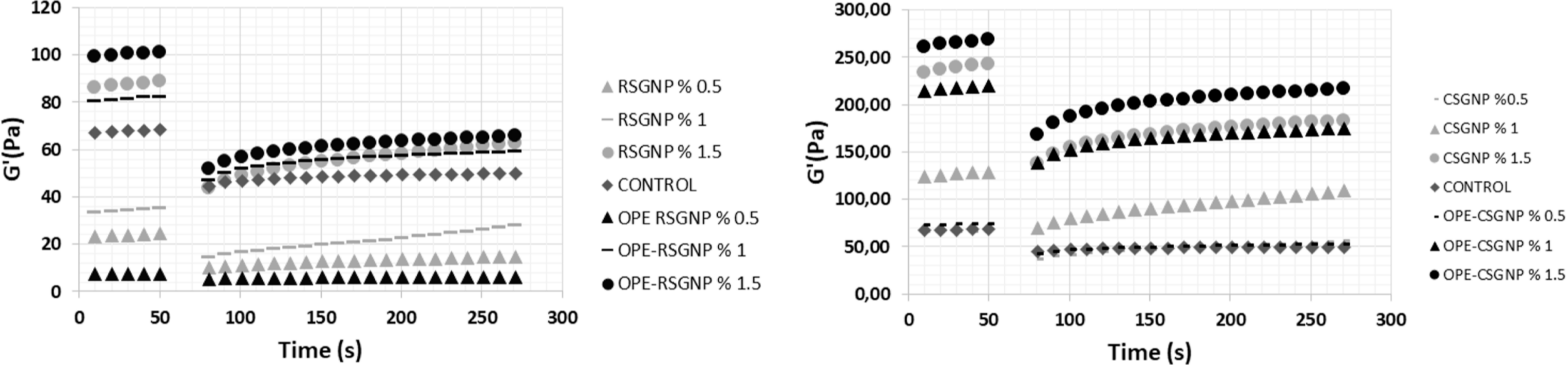
3-ITT of the storage modulus (*G*′) of the mayonnaise samples RSGNP: rocket seed gum nanoparticle, CSGNP: chia seed gum nanoparticle, OPE–RSGNP: olive pomace extract-loaded RSGNP, and OPE–CSGNP: olive pomace extract-loaded CSGNP.

The deformation of mayonnaise formed during the production process, as well as handling, transportation, storage, and consumption. 3-ITT tests were used to simulate the conditions of the production process for the food industry. The test provided information about the deformation and recovery of food materials to simulate and perform pumping and instant stirring operations.^4,25^ The viscoelastic properties of the mayonnaise samples like *G*′ values were higher than the *G*″ values, which means that solid-like property dominated mayonnaise properties. Therefore, thixotropic properties of the mayonnaise samples were observed only in terms of *G*′ values. The 3-ITT results of the mayonnaises deformed with different shear stress are presented in Figure 4, and the 3-ITT parameters are given in Table 5.

**Table 5:** Thixotropic Parameters of Mayonnaise Samples^a^

run	NP concentration (%)	*D*_r_ (%)	Rec_30_ (%)
RSGNP	0.5	57.29 ± 1.34a	48.36 ± 2.11e
RSGNP	1	56.52 ± 1.87a	52.13 ± 1.25d
RSGNP	1.5	49.46 ± 1.82b	58.78 ± 3.37c
OPE–RSGNP	0.5	54.21 ± 2.37a	60.98 ± 0.75c
OPE–RSGNP	1	41.16 ± 2.03c	65.84 ± 0.58b
OPE–RSGNP	1.5	43.92 ± 0.94c	66.29 ± 1.24b
CSGNP	0.5	41.10 ± 2.25c	58.97 ± 1.55c
CSGNP	1	43.38 ± 3.42c	66.37 ± 0.47b
CSGNP	1.5	40.91 ± 2.13c	67.86 ± 1.30b
OPE–CSGNP	0.5	41.48 ± 1.18c	65.29 ± 1.48b
OPE–CSGNP	1	35.08 ± 1.81d	73.34 ± 2.48a
OPE–CSGNP	1.5	35.25 ± 1.25d	73.56 ± 1.48a
control	0	33.81 ± 1.93d	70.60 ± 2.15ab

a RSGNP: rocket seed gum nanoparticle, CSGNP: chia seed gum nanoparticle, OPE–RSGNP: olive pomace extract-loaded RSGNP, and OPE–CSGNP: olive pomace extract-loaded CSGNP. Lowercase letters indicate the relationship between all mayonnaises. Values that do not share the same letter differ significantly (*p* < 0.05).

The control sample of the recovery percentage was found 70.60 ± 2.15%. The control mayonnaise of the recovery percentage was significantly higher than the RSGNP with all nanoparticle concentrations (OPE–RSGNP with 0.5% and CSGNP with 0.5% mayonnaise sample). As can be seen from the table, all of the OPE–CSGNP samples and CSGNP and OPE–RSGNP samples with 1 and 1.5% concentrations showed a similar recovery behavior to the control sample (*p* < 0.05). The high recovery properties of products such as mayonnaise and salad dressing are vital for the use of these products in a food application such as hamburgers and French fries.^4^ In addition, at least 70% of recovery percentage to have well thixotropic recovery mayonnaise samples had well thixotropic characteristics and have similar rheological properties, which are high viscoelasticity, consistency, and recovery properties. The consumption of the mayonnaise sample in the plastic bottle was imitated by 3-ITT parameters. The deformation of all mayonnaise samples was significantly higher than the control samples except OPE–CSGNP with 1 and 1.5% mayonnaise samples (*p* < 0.05). All emulsions exhibit thixotropic responses, which can confirm that the emulsions are shear-thinning pseudoplastic. A similar result was reported from previously published study.^45^ It was reported that novel mayonnaise samples Pickering stabilized by using apple pomace particles could be used as cholesterol-free mayonnaise.^4^ The thixotropic behavior was measured by 3-ITT with the *G*′ values. Their results displayed that micro jet and ultrasound novel mayonnaises exhibited a higher recovery rate than the control mayonnaise. Also, microjet novel mayonnaise displayed fast recovery than the ultrasound and high-speed shear homogenizer novel mayonnaise.

### 3.3 Emulsion Capacity and Stability

Emulsifying capacity and emulsion stability values are given in Table 2. As can be seen in Table 2, emulsifying capability increased with an increase in the nanoparticle concentration. This result could be related to more particles located into the oil droplets with an increase in the concentration.^30^ Also, the higher *K* value of the yolk-free mayonnaise samples led to increasing emulsion stability due to providing the highest viscosity with high-molecular weight and long-chain branch structure.^35,43^ Biopolymers stabilize droplets against coalescence, especially a combination of physical and chemical interactions, such as electrostatic and polymeric steric interactions, hydrogen bonding, hydrophobic association, and cation-mediated cross-linking.^1^ Gums also improve the technical and functional characteristics of emulsions such as aqueous solubility, thickening, gelling and gel stabilizing, and significantly sensory creation ability.^46^ Pickering emulsions which are stabilized by food-grade particles such as starch,^28^ apple pomace,^4^ and wheat gliadin^3^ implied the same order about particle size and concentration.

Emulsifying capacity was observed by using the creaming index as an indicator, and the emulsifying capacity values of the mayonnaise showed that an increase in the nanoparticle concentration with the smaller oil droplet size led to an increase in emulsifying capacity. The creaming effect is a unique property of the Pickering emulsions due to their larger droplets.^28,30^ The increasing concentration of nanoparticles caused the reduction in the creaming effect due to an increase in the surface coverage of the oil droplets. In addition, the association of the particles between droplets by aggregation of particles could inhibit the creaming effect.^27,28^ Similar results were reported in reference (28). (30),

### 3.4 Storage Stability

**Figure 5:**
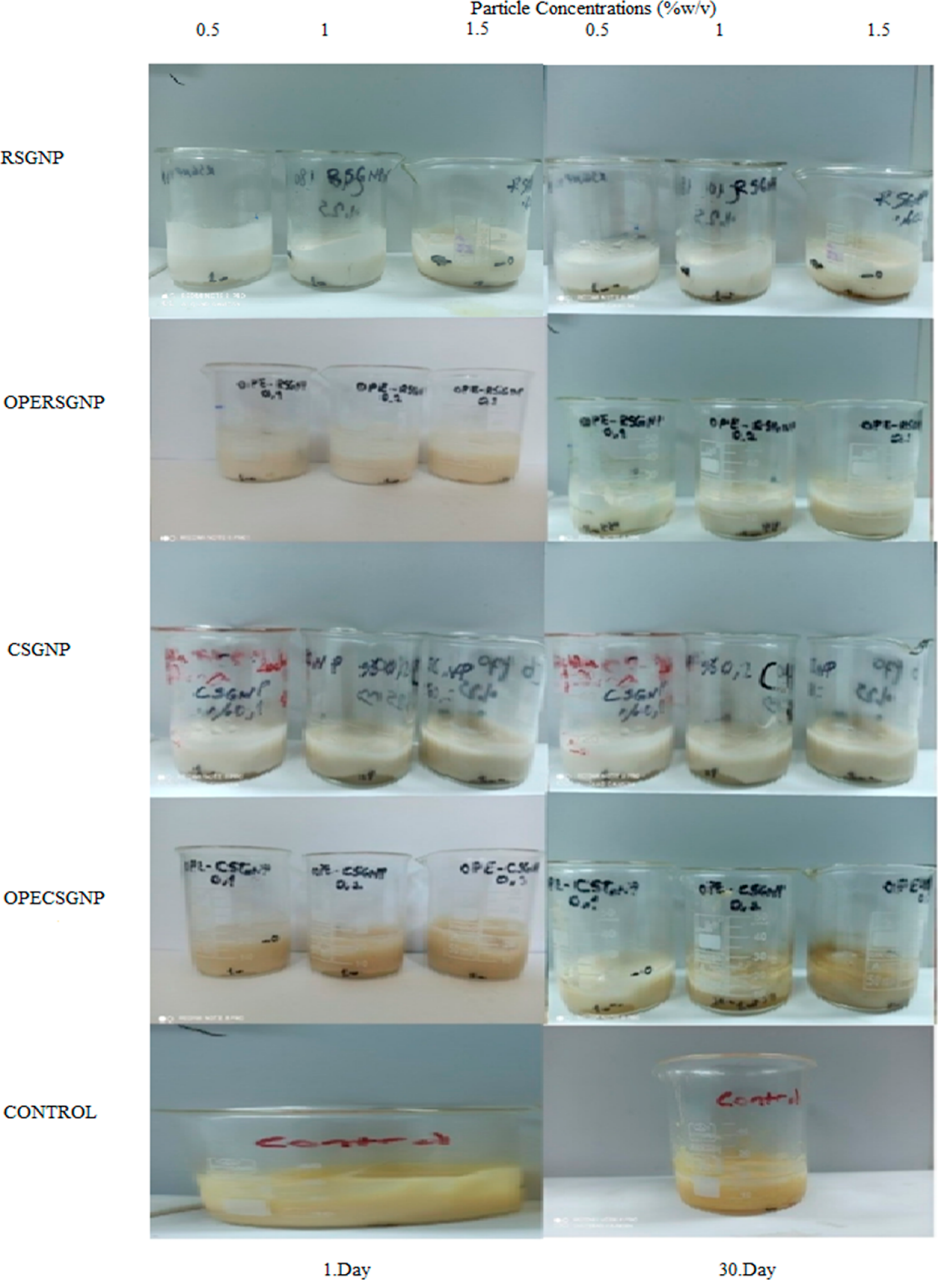
Appearance of the mayonnaise samples. RSGNP: rocket seed gum nanoparticle, CSGNP: chia seed gum nanoparticle, OPE–RSGNP: olive pomace extract-loaded RSGNP, and OPE–CSGNP: olive pomace extract-loaded CSGNP.

Emulsion appearance and mean droplet size were significant parameters for storage stability.^47^ The droplet size of the yolk-free mayonnaise samples with storage period time 1 day and 30 days is given in Table 2. The storage time slightly affects the droplet size of the yolk-free mayonnaise samples except for OPE–CSGNP at all concentrations. Our previous study showed that the particle size increased during the encapsulation of OPE in CSGNP. The higher molecular weight of the CSG than the RSG could also affect storage stability. In addition, the highest oil droplet size was seen at 0.5% concentrations of CSGNP. At these concentrations, lower viscosity was achieved, and oil droplets can easily move and coalesce in the continuous phase. However, the droplet size of the emulsion had decreased with an increase in concentrations. An increase in the nanoparticle concentration led to a decrease in the droplet size and approached the droplet size of the control mayonnaise. The smaller droplet size of the emulsion presented high stability. This situation could be explained with gel-like network which limited the movement of the oil droplets.^46^ According to Figure 5, there was no change in creaming behavior at 1 day and 30 day. This indicated that control and yolk-free mayonnaise samples had storage stability due to high viscosity and a strong network.

### 3.5 Oxidative Stability

Table 6 shows the IP (h) values of the collection emulsions. The IP values of the samples varied from 4.05 to 6.74 h and increased as increasing nanoparticle concentrations. As can be seen in Table 6, a significant difference was observed between the IP values of the samples. The IP value of OPE-loaded nanoparticles can be explained by the more effective scavenging of free radicals by the controlled release of charged phenolic compounds. IP values of control mayonnaise were found significantly higher than the blank gum nanoparticles. These results could be related to the antioxidant activity of egg yolk. Egg yolks are obtained from the light centrifugation of diluted egg yolk and are composed of 70% high-density lipoproteins (HDLs), 16% phosvitin, and 12% low-density lipoproteins (LDLs).^48^ The IP value of the control mayonnaise sample is due to the antioxidant properties of many egg proteins such as ovalbumin, ovotransferrin, phosvitin, and egg lipids such as phospholipids, as well as some micronutrients such as vitamin E, vitamin A, selenium, and carotenoids in the egg yolk.^49^ Hydroxyl amines in the side chains of phospholipids play a role in radical scavenging and show antioxidant properties.^50^ The unsaturated structure and aromatic carotenoid rings help neutralize singlet oxygen and free radicals and protect against oxidative damage.^51^ Phosvitin can increase the oxidation stability of lipids and proteins through its iron-chelating activities.^52^

**Table 6:** IP Values of Mayonnaise Samples^a^

samples	NP concentration (%)	IP (h)
RSGNP	0.5	4.05 ± 0.05h
RSGNP	1	4.35 ± 0.11g
RSGNP	1.5	4.55 ± 0.41f
OPE–RSGNP	0.5	5.58 ± 0.32c
OPE–RSGNP	1	6.18 ± 0.10b
OPE–RSGNP	1.5	6.74 ± 0.25a
CSGNP	0.5	4.12 ± 0.51g
CSGNP	1	4.52 ± 0.07f
CSGNP	1.5	4.85 ± 0.09e
OPE–CSGNP	0.5	5.75 ± 0.10c
OPE–CSGNP	1	6.05 ± 0.12b
OPE–CSGNP	1.5	6.53 ± 0.37a
control	0	5.05 ± 0.11d

a RSGNP: rocket seed gum nanoparticle, CSGNP: chia seed gum nanoparticle, OPE–RSGNP: olive pomace extract-loaded RSGNP, and OPE–CSGNP: olive pomace extract-loaded CSGNP. Lowercase letters indicate the relationship between all mayonnaises. Values that do not share the same letter differ significantly (*p* < 0.05).

However, IP values of OPE-loaded RSGN and CSGNP-stabilized egg yolk-free mayonnaise samples increased with increasing gum nanoparticle concentrations. Also, IP values of these mayonnaise samples were found significantly higher than the control mayonnaise sample due to the increasing amount of OPE in egg yolk-free mayonnaise samples. The results show that OPE-loaded nanoparticles slow down the oxidation of egg yolk-free Pickering emulsions. This can be explained by the localization of OPE phenolic compounds instead of egg yolk powder at the oil-in-water interface of Pickering emulsions. The interaction of OPE phenolic compounds with other antioxidant compounds may have enhanced antioxidant activity and led to higher IP values.^19,21,53^ Moreover, nano-encapsulated OPE can be used as an alternative to providing oxidative stability of Pickering emulsions instead of egg yolk since the degradation of nanoencapsulation of OPE phenolic compounds is prevented by using natural gums as wall materials for nanoencapsulation. The combination of the emulsion-based encapsulation technology and antioxidant enrichment can provide synergistic effects of oxidative stability to many products.

### 3.6 Sensory Properties of the Mayonnaise Samples

The results of the statistical analysis of all mayonnaise applications for appearance, taste, color, spreadability, texture, and overall acceptability are given in Table 7. According to the table, the sensory quality criteria of the yolk-free mayonnaise samples, except for the color and appearance characteristics, showed a sensory score close to the control samples. While no statistical difference was observed in the appearance properties with the control mayonnaise sample at low nanoparticle concentrations (0.5–1%), the appearance scores of the samples containing 1.5% nanoparticles were lower than the control sample. The control mayonnaise sample has a yellowish color because it contains egg yolk. Egg yolk-free samples are lighter in color than the control sample. The main reason for the decrease in the scores in the appearance quality criterion may be the lightning of the colors of the samples by removing the egg yolk from the formulation. However, there is a dark greenish appearance in the color values of the samples prepared with high 1.5% OPE-loaded nanoparticles. A statistical decrease was observed in the sensory scores of the samples containing 1.5% OPE-loaded nanoparticles in the taste properties of the samples. In this case, the bitter taste of olive waste phenolic may have been perceived negatively by the panelists. There was no negative difference in spreadability and texture values of the samples compared to the control sample. These results are in agreement with the rheology results. When we examined the general taste scores, no significant difference was observed between the sensory scores of the samples prepared with OPE-loaded nanoparticles and the sensory scores of the control sample. These results indicated that OPE-loaded nanoparticles would not pose a problem in terms of sensory quality of the mayonnaise sample with the improvement in color properties.

**Table 7:** Sensory Properties of Mayonnaise Samples^a^

run	NP concentration (%)	appearance	taste	color	spreadability	texture	overall acceptability
RSGNP	0.5	4.36 ± 0.25a	4.30 ± 0.33a	4.23 ± 0.26a	4.26 ± 0.13a	4.33 ± 0.30a	4.30 ± 0.20a
RSGNP	1	4.03 ± 0.28ab	4.06 ± 0.13a	4.13 ± 0.17a	4.03 ± 0.15a	4.40 ± 0.16a	4.23 ± 0.18a
RSGNP	1.5	4.36 ± 0.31a	4.16 ± 0.24a	3.96 ± 0.15ab	4.13 ± 0.23a	4.00 ± 0.12ab	4.33 ± 0.31a
OPE–RSGNP	0.5	3.90 ± 0.16ab	4.06 ± 0.29a	3.83 ± 0.19a	4.40 ± 0.16a	4.26 ± 0.28a	4.06 ± 0.23ab
OPE–RSGNP	1	4.23 ± 0.12a	4.10 ± 0.18a	4.26 ± 0.18a	4.30 ± 0.25a	3.96 ± 0.21ab	3.94 ± 0.16ab
OPE–RSGNP	1.5	3.49 ± 0.23b	3.76 ± 0.14b	4.17 ± 0.23a	3.53 ± 0.37ab	3.70 ± 0.27ab	3.93 ± 0.12ab
CSGNP	0.5	4.33 ± 0.20a	4.16 ± 0.39a	4.33 ± 0.20a	4.36 ± 0.26a	4.20 ± 0.25a	4.03 ± 0.21ab
CSGNP	1	3.86 ± 0.22ab	3.73 ± 0.18ab	3.96 ± 0.14a	4.02 ± 0.16ab	4.03 ± 0.19ab	3.90 ± 0.20ab
CSGNP	1.5	3.46 ± 0.17b	3.46 ± 0.17b	3.46 ± 0.16b	3.96 ± 0.19ab	3.80 ± 0.23a	4.13 ± 0.25a
OPE–CSGNP	0.5	3.63 ± 0.15b	4.16 ± 0.30a	4.03 ± 0.18a	3.93 ± 0.18ab	4.00 ± 0.15a	4.06 ± 0.28ab
OPE–CSGNP	1	3.86 ± 0.24ab	3.86 ± 0.26ab	3.93 ± 0.15a	3.76 ± 0.24ab	3.93 ± 0.21ab	3.80 ± 0.16ab
OPE–CSGNP	1.5	3.53 ± 0.14b	3.71 ± 0.20ab	3.46 ± 0.13b	3.23 ± 0.57ab	3.46 ± 0.14b	3.73 ± 0.14ab
control	0	4.20 ± 0.15a	4.10 ± 0.24a	4.30 ± 0.26a	4.33 ± 0.29a	4.26 ± 0.22a	4.35 ± 0.18a

a RSGNP: rocket seed gum nanoparticle, CSGNP: chia seed gum nanoparticle, OPE–RSGNP: olive pomace extract-loaded RSGNP, and OPE–CSGNP: olive pomace extract-loaded CSGNP. Lowercase letters indicate the relationship between all mayonnaises. Values that do not share the same letter differ significantly (*p* < 0.05).

## 4 Conclusions

The yolk-free mayonnaise was made by gum nanoparticles with or without loading OPEs. The results showed that the appearance of the mayonnaise samples had no significant changes except the color. The droplet size of the yolk-free mayonnaise samples decreased by increasing the nanoparticle concentration. OPE–RSGNP with 1.5% had the smallest droplet size and was found lower than the control mayonnaise. The emulsion stability and capacity of the yolk-free mayonnaise samples were found similar to the control samples. The yolk-free mayonnaise samples showed pseudoplastic behavior with solid-like properties. All mayonnaise samples of the *G*′ values were found higher than the *G*″ values, which means that solid-like properties dominate the viscous properties of the mayonnaise samples. In terms of recovery, no significant changes were observed between the OPE–RSGNP and CSGNP with 1 and 1.5%, and OPECSGNP with all concentrations of the mayonnaise samples and the control mayonnaise (*p* < 0.05) and showed similar thixotropic properties. Thus, these findings showed that the gum nanoparticles could be used as an alternative to the egg yolk in conventional mayonnaise. Further studies are recommended to decrease droplet diameter and showed textural and tribological properties to optimize yolk-free mayonnaise.
